# Longevity of Amalgam Versus Composite Resin Restorations in Permanent Posterior Teeth: A Systematic Review

**DOI:** 10.7759/cureus.88836

**Published:** 2025-07-27

**Authors:** Sumita Bhagwat, Lalitagauri Mandke, Mansi Vandekar, Nidhi Basmatkar, Aishwarya Pawar, Rajni Khatri

**Affiliations:** 1 Department of Conservative Dentistry and Endodontics, D.Y. Patil University School of Dentistry, Navi Mumbai, IND

**Keywords:** amalgam, composite resin, dental restorations, longevity, posterior teeth, systematic review

## Abstract

The present systematic review aims to compare the longevity of amalgam and composite resin restorations in adult human posterior permanent teeth, evaluating clinical performance, survival rates, failure causes, and influencing factors. Following Preferred Reporting Items for Systematic Reviews and Meta-Analyses (PRISMA) guidelines, a comprehensive search was conducted in PubMed, Scopus, Web of Science, and other databases. Studies were included if they assessed the longevity of amalgam and composite resin restorations in adult posterior teeth with at least one year of follow-up. The Newcastle-Ottawa Scale (NOS) and Cochrane Risk of Bias (ROB) tool were used for the assessment of risk of bias. Eight studies (2003-2023) met the inclusion criteria, comprising randomized clinical trials, prospective, retrospective, and cross-sectional studies. Amalgam restorations exhibited superior longevity, with median survival times exceeding 16 years, compared to 11 years for composite restorations. Secondary caries was the most common cause of composite failure, whereas fracture was the primary reason for amalgam replacement. Patient factors, including oral hygiene and bruxism, significantly influenced restoration longevity. Amalgam restorations demonstrate greater durability than composite resins in posterior teeth. However, aesthetic preferences and advancements in composite materials continue to drive their usage. Future research should focus on improving composite longevity to provide viable alternatives to amalgam.

## Introduction and background

Dental restorations have long been a cornerstone of modern dentistry, essential for preserving and restoring the structural integrity and functionality of teeth affected by caries, trauma, or various other dental conditions. Among the multitude of materials employed for this purpose, dental amalgam and composite resin have stood out as two of the most prevalent choices [1]. The selection of restorative materials has been widely studied, with longevity being a key factor in evaluating their clinical effectiveness.

Amalgam, composed primarily of a mixture of metals, including silver, tin, copper, and mercury, has been in use for over a century [2]. Its long-standing popularity can be attributed to its durability, cost-effectiveness, and ease of manipulation, making it a primary choice for restoring posterior permanent teeth. In contrast, composite resin, a tooth-colored material, has garnered increased favor due to its aesthetic properties and its capacity to bond directly to the tooth structure [3]. The longevity of restorations, whether amalgam or composite resin, is important in assessing clinical success, as the ability to withstand challenges posed by masticatory forces, temperature fluctuations, and the oral environment is considered a fundamental benchmark in dental practice [4].

In posterior permanent teeth, these factors are more significant due to higher occlusal loads and complex functional demands, making it crucial to understand the comparative longevity of amalgam and composite resin restorations. This systematic review aims to analyze the existing literature on the topic, combining data from various clinical studies and exploring several relevant aspects, including clinical performance, success rates, longevity, and potential factors influencing the outcomes of amalgam and composite resin restorations. The findings of the present review will serve as a foundation for future research directions and clinical practice improvements, promoting a holistic understanding of the complex factors that influence the durability of dental restorations.

## Review

Methodology

The present systematic review was conducted following a comprehensive and structured methodology to assess the longevity of amalgam and composite resin restorations in human posterior permanent teeth. The research design adhered to the Preferred Reporting Items for Systematic Reviews and Meta-Analyses (PRISMA) guidelines, and the protocol was registered in the International Prospective Register of Systematic Reviews (PROSPERO) (Reference ID: CRD42023469680) [5].

Search Strategy and Inclusion Criteria

To identify relevant studies, a systematic search strategy was employed. Databases including PubMed, Scopus, Web of Science, EBSCOhost (EBSCO Information Services, MA, USA), ScienceDirect, and Google Scholar were searched using predefined search terms and Medical Subject Headings (MeSH) as appropriate. The search strategy included a combination of keywords such as "amalgam," "composite resin," "dental restorations," "posterior teeth," and "longevity." The search was limited to articles published in the English language from the inception of the databases until March 2025. A sample search string in the PubMed database is demonstrated in Appendix 1.

Studies were included if they reported on the longevity of amalgam and composite resin restorations in human posterior permanent teeth, involved human subjects, were longitudinal with at least one month of follow-up, and were published as full-text articles in English in peer-reviewed journals. Studies were excluded if they did not meet these criteria or if they were review articles, case reports, or conference abstracts. Two independent reviewers performed the initial screening of titles and abstracts identified through the search strategy. After the initial screening, full-text articles that met the inclusion criteria were obtained for further assessment. Any discrepancies between the reviewers were resolved through discussion or consultation with a third reviewer.

Data Extraction

Data from the selected studies were extracted using a predefined form, collecting information on study characteristics (e.g., author(s), publication year, study design), participant characteristics (e.g., sample size, age), intervention details (e.g., type of restorative material, technique), outcome measures (e.g., survival rates, reasons for failure), and follow-up duration.

Assessment of Risk of Bias

The quality of observational studies included in this review was assessed using the Newcastle-Ottawa Scale (NOS), a Cochrane-endorsed tool for evaluating non-randomized studies [6]. Two authors independently conducted the appraisal, and any disagreements were resolved through consultation with a third reviewer. For interventional studies, the Cochrane Risk of Bias version 2 (ROB-2) tool was employed [7]. Visual representation of the domain-level assessments was generated using RevMan software version 5.3 (The Cochrane Collaboration, London, UK) [7]. Narrative synthesis was conducted for the included studies, and where feasible, a meta-analysis was performed based on data availability and study homogeneity.

Statistical Analysis

Risk ratios (RR) with corresponding 95% confidence intervals (CI) were calculated for binary outcomes. A fixed-effect model was used when heterogeneity was not evident (p > 0.05 or I² ≤ 24%), while a random-effects model was applied when heterogeneity was present. All analyses were conducted using RevMan version 5.3, with statistical significance set at p < 0.05. To evaluate inconsistencies among study findings, Cochran’s Q test and the I² statistic were applied. Heterogeneity was deemed statistically significant at p < 0.1. According to the Cochrane Handbook, I² values between 0-40% suggest low heterogeneity, 30-60% indicate moderate heterogeneity, 50-90% substantial heterogeneity, and values exceeding 75% indicate considerable heterogeneity. Potential publication bias was examined using Begg’s funnel plot, which graphically displays the relationship between study effect sizes and their standard errors. Any asymmetry observed in the funnel plot was interpreted with caution, as it may reflect bias, small-study effects, or genuine associations between study size and effect magnitude.

Results

**Figure 1:**
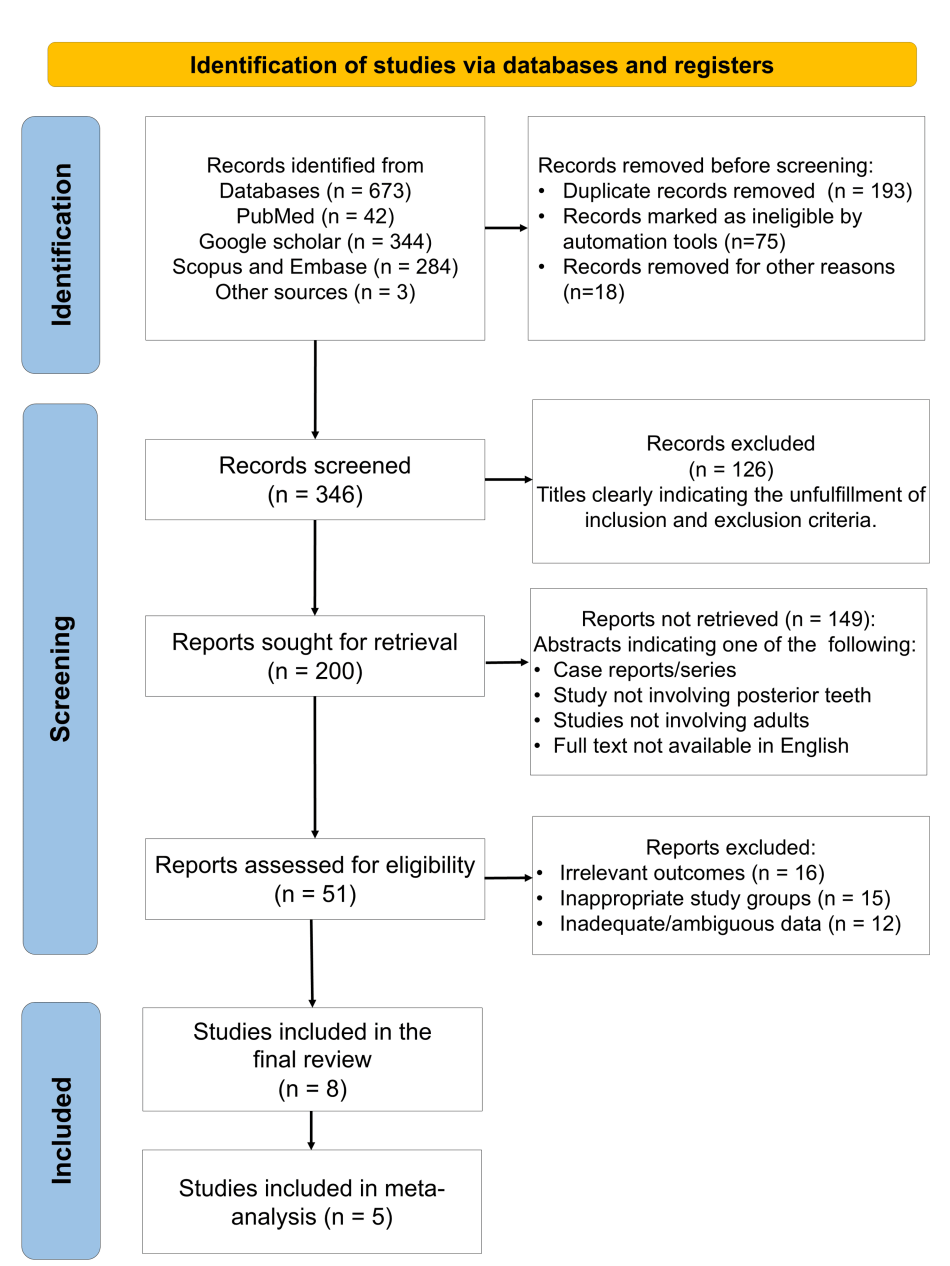
PRISMA flow diagram indicating the selection process of the articles in the present systematic review PRISMA: Preferred Reporting Items for Systematic Reviews and Meta-Analyses

A total of N = 673 studies were identified after a search across all databases, out of which 346 were selected following the removal of duplicates. About n = 200 reports were sought for retrieval, and after screening the abstracts for (Population, Intervention, Comparison, Outcome, Study Design) PICOS criteria, only 51 full-text reports were assessed for eligibility. Finally, eight studies (Figure 1) were included in the data synthesis, spanning from the year 2003 to 2023.

Of these, two were randomized clinical trials, two were prospective studies, three were retrospective studies, and one was a cross-sectional study [8-15]. The data extracted from these articles is summarized in Table 1.

**Table 1:** Data extracted related to the characteristics and outcomes of the studies included in the present systematic review AM: amalgam; CR: composite resin; AFR: annual failure rate; NP: not provided; ADA: American Dental Association

Sr. No.	Author	Sample size	Age of sample population	Restorations	Follow-up period	Amalgam failure	Composite failure	Comparison
1	Nieuwenhuysen et al. (2003) [8]	811	16–80 years (Median: 40)	722 AM, 115 CR	Every 4 years for ~20 years, then 3 years	Not specified	Not specified	Fracture was the leading failure reason, particularly in CR premolars (18%). Secondary caries affected AM molars the most (6%). Median survival: 16+ years (AM), 11 years (composite). Extensive AM restorations may serve as crown alternatives.
2	Bernardo et al. (2007) [9]	472	8–12 years	239 AM, 233 CR	7 years	Not provided	Composite showed 3.5x higher risk of secondary caries	AM showed higher survival (94.4%) than composite (85.5%). Failure mainly due to secondary caries. Annual failure rates were lower for amalgam.
3	Opdam et al. (2007) [10]	2867	NP	912 AM, 1255 CR	5, 10 years	Caries (29%), fracture (17%), endo issues (13%)	Caries (38%), fracture (11%), endo issues (10%)	Failure: 182 AM, 259 CR. 5-year survival: 89.6% (AM), 91.7% (CR); 10-year: 79.2% (AM), 82.2% (CR).
4	Opdam et al. (2010) [11]	1949	NP	1202 AM, 747 CR	5 and 12 years	Fracture (5.9%), secondary caries (5.7%)	Secondary caries (6.6%)	293 AM and 114 CR failed. At 5 years, performance was similar; at 12 years, CR had better survival. AFR for CR declined over time, while it increased for AM.
5	McCracken et al. (2013) [12]	6218	NP	1940 AM, 3892 CR	6-monthly over 5 years	Odds ratio 1	Odds ratio 1.1	Failure rate for both groups was ~6% during follow-up.
6	Kemaloglu et al. (2016) [13]	25	18–60 years	25 AM, 25 CR	2 weeks to 3 years	Not provided	Post-op sensitivity decreased significantly over time	At 3 years, all restorations met ADA minimal standards. Slightly more sensitivity observed in AM, though not statistically significant.
7	Al-Asmar et al. (2023) [14]	297	>18 years (Mean: 37.43)	261 AM, 57 CR	9 months	Mostly due to aesthetics, caries, or fracture	Commonly due to caries, discoloration, and aesthetics	Majority of replaced amalgams were >10 years old; CR were more often replaced in poor hygiene cases. Bruxism patients were more likely to have AM restorations replaced.
8	Santos et al. (2023) [15]	110	26–86 years (Mean: 65.5)	69 AM, 50 CR	5 years	Caries (10.1%), poor margins (8.7%), tooth fracture (7.2%)	Restoration fracture (16%), poor margins (8.2%)	At 5 years, 76.8% AM and 78% CR were satisfactory. Caries are more common in AM; fractures and poor anatomy are more frequent in CR. No significant survival difference.

The sample size across these studies had a wide range, from 25 to 6218. McCracken et al. conducted an extensive prospective study on 1940 amalgam and 3892 composite resin restorations [12]. The number of amalgam and composite resin restorations was nearly equal in only two studies, while all other studies displayed a wide imbalance between the two types of restorations, with one type significantly outnumbering the other. The inclusion criteria across various studies included patients only in need of at least two Class II restorations in molars and premolars, asymptomatic teeth, occlusal and adjacent teeth in contact, and cavity sizes exceeding one-third of the faciolingual cusp distance. Bernardo et al. (2007) included children with at least one carious lesion in a permanent posterior tooth, no prior amalgam exposure, urinary mercury <10 µg/L, blood lead <15 µg/dL, IQ ≥67 (Comprehensive Test of Nonverbal Intelligence), and no interfering health conditions [9].

Santos et al. (2023) included patients treated at the school’s dental clinic five years ago with complex amalgam or resin composite restorations (≥3 surfaces) on molars or premolars by dental students [15]. Only three studies specified exclusion criteria: fewer than 20 teeth, poor oral hygiene, bruxism, periodontitis, allergic reactions to materials, and restorations with a glass-ionomer liner. Opdam et al. (2010) observed that before 1994, most large Class II cavities were restored with amalgam, while after 1995, amalgam was no longer used [11]. A transition to composite occurred in 1994-1995, so restorations from this period were excluded to avoid confounding by material selection. Dispersed alloy and hybrid composite were used in most of the studies. The restorations were performed uniformly by a fixed number of experienced dentists in n = 3 studies, by dentists of varied experience in two studies, and by undergraduate dental students in one study. The use of local anesthesia and isolation with a rubber dam, cotton rolls, and saliva ejector was mentioned as a common protocol for both types of restorations in two studies.

Amalgam restoration protocol involved cavity preparation, use of retentive features (pins if needed), calcium hydroxide liners for deep cavities, metal matrix placement with sycamore wooden wedges, and polishing after 48 hours with pointed stones and a rubber cup. The composite restoration protocol included matrix placement (circumferential/sectional with an elastic ring), etching with 37% orthophosphoric acid (15 s), bonding agent application, resin composite placement, light curing as per manufacturer’s instructions, and finishing with fine diamond burs and rubber points. Restoration longevity was assessed based on functional ability or United States Public Health Service (USPHS) criteria [16]. Follow-up periods varied, ranging from two weeks to three years in randomized clinical trials and nine months to 12 years in other studies.

Al-Asmar et al. (2023) found that aesthetic demand was the primary reason for replacing amalgam restorations [14]. Class II restorations that included one or two proximal walls; secondary caries was the main cause. Tooth fractures occurred in only two molars (Cl I and mesial-occlusal-distal (MOD)), while restoration fractures were most common in Class II molars (36 cases). Of the 19 painful amalgam restorations, three required occlusal adjustment, five were referred for endodontic treatment, and 11 were replaced with a liner or base. Proximal defects in amalgam restorations were more often overhangs than open contacts. Secondary caries and marginal discoloration (n = 15) were the most common reasons for replacing or repairing resin composite restorations. Patients who had teeth with marginal discoloration sought replacement due to fear of secondary caries or for aesthetic reasons. Bernardo et al. (2007) reported that the risk of secondary caries was 3.5 times greater in the composite group [9]. Nieuwenhuysen et al. (2003) found that restoration fracture was the most common reason for failure (8%), particularly in composite restorations on premolars (18%). Secondary caries (6%) was the next most frequent cause, primarily affecting complete amalgam restorations in molars [8]. The median survival times exceeded 16 years for amalgam restorations and 11 years for the resin restorations; extensive amalgam restorations, but not composite resin restorations, can be used as an appropriate alternative to crowns, considering the longevity of the restorations [8]. Bernardo et al. (2007) reported a 94.4% survival rate for amalgam restorations and 85.5% for composites. Annual failure rates ranged from 0.16-2.83% for amalgam and 0.94-9.43% for composite, with secondary caries as the primary cause of failure for both [9]. In contrast, McCracken et al. (2013) found an equal annual failure rate of 6% for both materials [12].

Al-Asmar et al. (2023) found that 56% of replaced amalgam restorations were over 10 years old, compared to 32% for composite resin. Poor oral hygiene was linked to more composite replacements (37%) than amalgam (28%), while bruxism and cuspal attrition were associated with more amalgam replacements (26%) than composite (5%). Bruxism and cuspal attrition were diagnosed in 4% of replaced amalgam and 5% of replaced composite restorations [14].

Santos et al. (2023) retrospectively assessed 119 restorations, finding 76.8% of amalgam and 78.0% of resin composite restorations satisfactory. Secondary caries was more common in complex Class II amalgam restorations, while composite restorations had higher rates of fracture and poor anatomy. The five-year survival rates showed no significant difference between the two materials (76.8% vs. 78%) [15].

**Figure 2:**
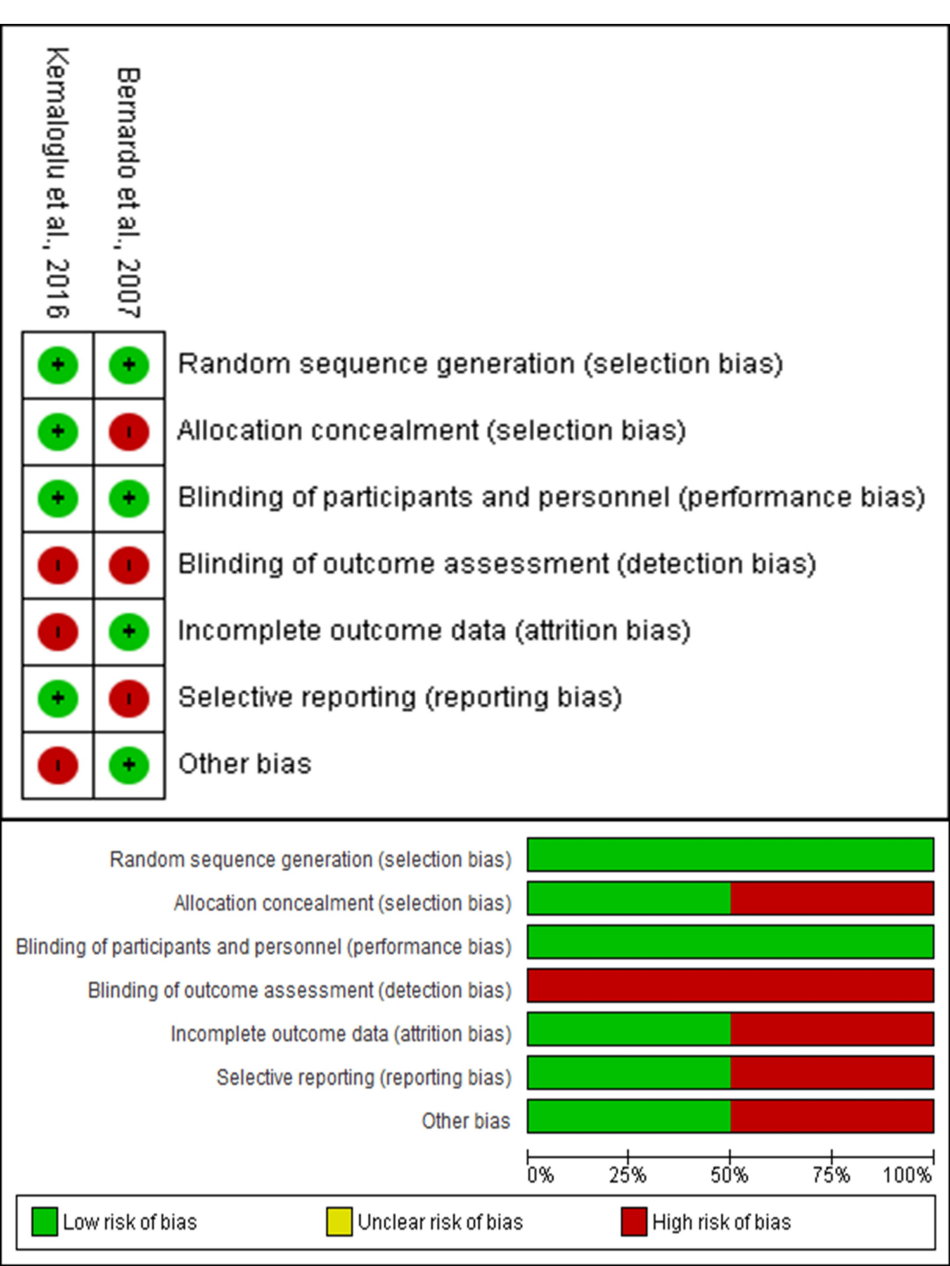
Risk of bias assessment of the included clinical studies using the Cochrane Risk of Bias-2 tool [9,13]

The included studies were comparable in methodological quality, with a moderate to high risk of bias across all domains. The highest risk was observed in outcome assessment blinding (detection bias), while the lowest was in random sequence generation (selection bias). Other domains showed a moderate risk of bias. The Cochrane ROB-2 tool assessment is depicted in Figure 2.

Among the included prospective/retrospective observational and cross-sectional studies, it did not reach the maximum score of the NOS [6]. One study (Opdam et al., 2010) had the highest score in selection criteria, all the included studies had the maximum score in the comparability outcome, and they were considered to have the highest level of quality with an estimated low risk of bias [11]. For exposure criteria, one study (Al-Asmar et al., 2023) reported a high risk of bias, as the study had a moderate to low risk of bias, with the overall quality of the study being good [14]. The risk of bias of the included study through the NOS is depicted in Table 2.

**Table 2:** Risk of bias in the cross-sectional and retrospective studies assessed using the Newcastle-Ottawa Scale * corresponds to one score

Author, year	Selection (Max = 4)	Comparability (Max = 2)	Exposure (Max = 3)	Overall quality score (Max = 9)
Nieuwenhuysen et al., 2003 [8]	**	**	**	6
Opdam et al., 2007 [10]	***	**	***	8
Opdam et al., 2010 [11]	****	**	**	8
McCracken et al., 2013 [12]	***	**	***	8
Al-Asmar et al., 2023 [14]	**	**	*	5
Santos et al., 2023 [15]	**	**	**	6

Synthesis of results

The RR is used as a summary statistic measure for dichotomous outcomes. The outcomes were assessed in terms of better effectiveness between amalgam and composite restoration in terms of secondary caries, restoration fracture, tooth fracture, and aesthetics.

Secondary Caries

**Figure 3:**
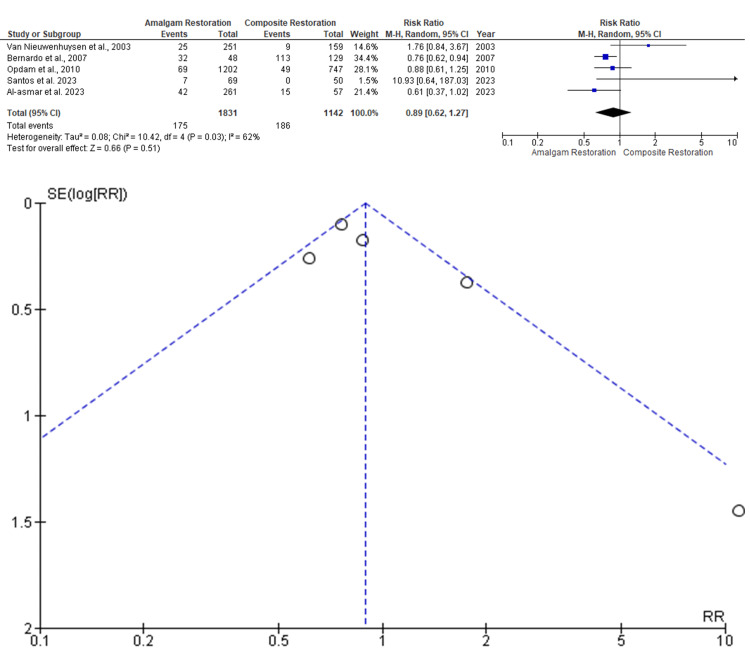
Forest plot showing comparison of secondary caries rate between amalgam and composite restorations, and funnel plot showing asymmetric distribution with absence of systematic heterogeneity [8,9,11,14,15]

Data was evaluated from five studies from an aggregate of 2973 posterior teeth, of which 1831 teeth were evaluated by amalgam restoration and 1142 teeth were evaluated by composite restoration for the evaluation of the better effectiveness between the two restorative materials in terms of secondary caries as an outcome. As shown in Figure 3, the RR is 0.89 (0.62 - 1.27), and the pooled estimates favor amalgam restoration. This signifies that the secondary caries rate on average was 0.89 times higher in amalgam restoration compared to the composite restoration, and this difference is not statistically significant (p>0.05). Among the included studies, the highest weightage was given by Bernardo et al. 2007, while the lowest weightage of the study was given by Santos et al. 2023 [9,15]. The funnel plot did not show significant asymmetry, indicating the absence of publication bias.

Restoration Fracture

**Figure 4:**
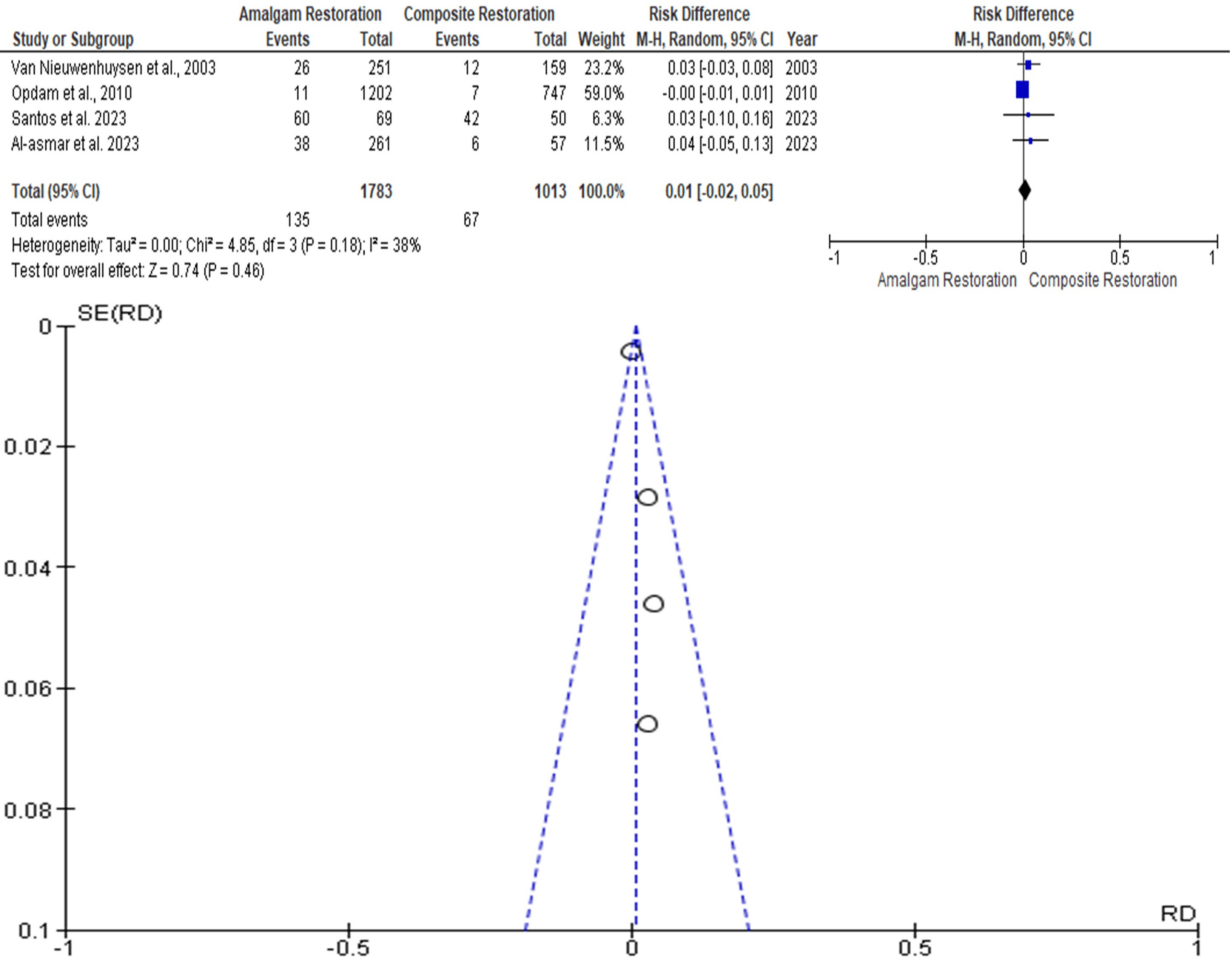
Forest plot showing comparison of restoration fracture of amalgam and composite restorations, and funnel plot showing asymmetric distribution with absence of systematic heterogeneity [8,11,14,15]

Data was evaluated from four studies from an aggregate of 2796 posterior teeth, of which 1783 teeth were evaluated by amalgam restoration and 1013 teeth were evaluated by composite restoration for the evaluation of the better effectiveness between the two restorative materials in terms of restoration fracture as an outcome. As shown in Figure 4, the RR is 0.01 (-0.02 - 0.05), and the pooled estimates favor composite restoration. This signifies that restoration fracture, on average, was 0.01 times more in composite restoration compared to the amalgam restoration, and this difference is not statistically significant (p>0.05). Among the included studies, the highest weightage was given by Opdam et al. 2010, while the lowest weightage of the study was given by Santos et al. 2023 [11,15]. The funnel plot did not show significant asymmetry, indicating the absence of publication bias.

Tooth Fracture

**Figure 5:**
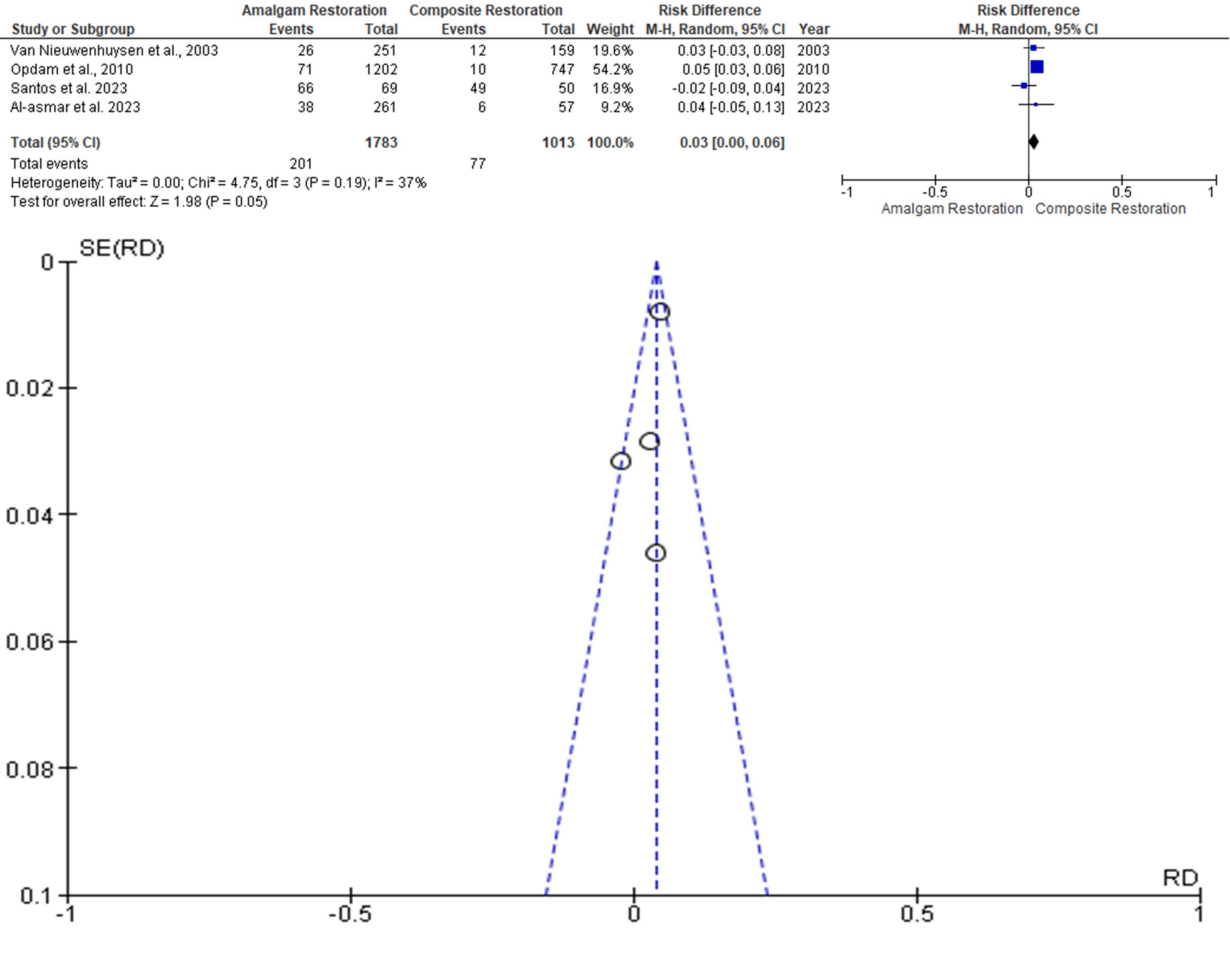
Forest plot showing comparison of tooth fractures between amalgam and composite restoration with regard to tooth fracture, and funnel plot showing asymmetric distribution with the absence of systematic heterogeneity [8,11,14,15]

Data was evaluated from four studies from an aggregate of 2796 posterior teeth, of which 1783 teeth were evaluated by amalgam restoration and 1013 teeth were evaluated by composite restoration for the evaluation of the better effectiveness between the two restorative materials in terms of tooth fracture as an outcome. As shown in Figure 5, the RR is 0.03 (0.00 - 0.06), and the pooled estimates favor composite restoration. This signifies that tooth fracture on average was 0.03 times more likely in composite restoration compared to the amalgam restoration, and this difference is not statistically significant (p>0.05). Among the included studies, the highest weightage was given by Opdam et al. 2010, while the lowest weightage of the study was given by Al-Asmar et al. 2023 [11,14]. The funnel plot did not show significant asymmetry, indicating the absence of publication bias.

Aesthetics

**Figure 6:**
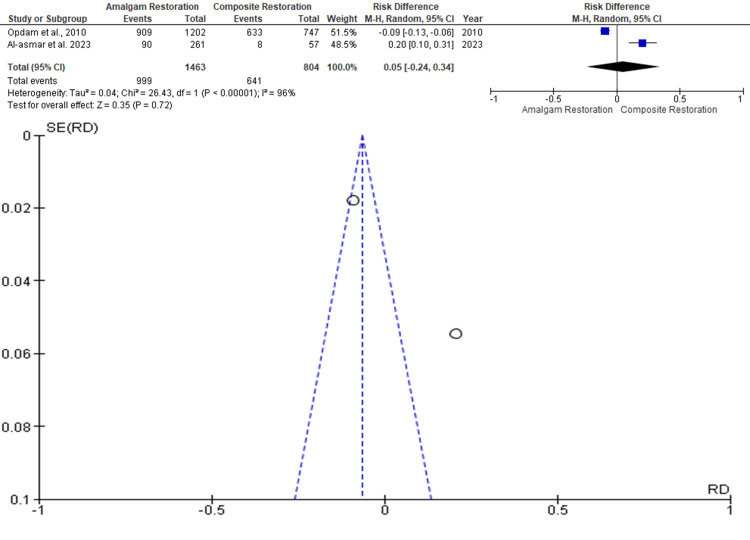
Forest plot showing comparison of aesthetics between amalgam and composite restoration with regard to tooth fracture, and funnel plot showing asymmetric distribution with absence of systematic heterogeneity [11,14]

Data was evaluated from two studies from an aggregate of 2267 posterior teeth, of which 1463 teeth were evaluated by amalgam restoration and 804 teeth were evaluated by composite restoration for the evaluation of the better effectiveness between the two restorative materials in terms of better aesthetics as an outcome. As shown in Figure 6, the RR is 0.05 (-0.24 - 0.34) and the pooled estimates favour composite restoration. This signifies that better aesthetics on average were 0.05 times more in composite restoration compared to the amalgam restoration, and this difference is not statistically significant (p>0.05). Among the included studies, the highest weightage was given by Opdam et al. 2010 while the lowest weightage of the study was given by Al-Asmar et al. 2023 [11,14]. The funnel plot did show significant asymmetry, indicating the presence of publication bias.

Discussion

The findings of this systematic review provide critical insights into the longevity of amalgam and composite resin restorations in human posterior permanent teeth. A comprehensive analysis of eight included studies revealed notable differences in the survival rates, failure modes, and risk factors associated with both restorative materials. These findings have important implications for clinical decision-making, particularly in selecting the most durable material for posterior restorations.

The results from multiple studies indicate that amalgam restorations generally demonstrate superior longevity compared to composite resin restorations. Studies such as those by Bernardo et al. (2007) and Nieuwenhuysen et al. (2003) reported significantly longer median survival times for amalgam restorations, often exceeding 16 years, whereas composite resin restorations had a median survival time of approximately 11 years. The higher annual failure rates observed for composite resin restorations (ranging from 0.94% to 9.43%) compared to amalgam (0.16% to 2.83%) further substantiate the superior durability of amalgam restorations [8,9]. However, McCracken et al. (2013) reported a similar failure rate of 6% for both materials, suggesting that improvements in composite resin formulations and adhesive techniques may be narrowing the longevity gap [12].

The most commonly reported cause of failure for both types of restorations was secondary caries [17]. Bernardo et al. (2007) found that the risk of secondary caries was 3.5 times greater in composite restorations than in amalgam restorations, corroborating findings from other studies. This increased susceptibility to caries in composite restorations may be attributed to factors such as polymerization shrinkage, microleakage, and inadequate marginal adaptation, which facilitate bacterial infiltration. Conversely, the most frequent reason for amalgam restoration replacement was fracture, often associated with extensive Class II restorations or bruxism-related stress [9,18-20].

Another major cause of failure for composite restorations was material fracture, particularly in premolars, as reported by Nieuwenhuysen et al. (2003). In contrast, amalgam restorations were more frequently replaced due to aesthetic concerns rather than functional failure [8]. Al-Asmar et al. (2023) noted that aesthetic demand was a significant factor in the replacement of amalgam restorations, particularly in premolars and MOD restorations on molars. This highlights the growing patient preference for tooth-colored restorations despite the demonstrated durability of amalgam [14].

The influence of patient-related factors on restoration longevity was also evident across studies. Poor oral hygiene was more commonly associated with failure of composite restorations than amalgam restorations [21]. Al-Asmar et al. (2023) reported that patients with poor oral hygiene had a higher frequency of composite resin restoration failures (37%) compared to amalgam (28%). This could be due to the increased plaque accumulation around composite restorations, exacerbating the risk of secondary caries [22]. On the other hand, bruxism and cuspal attrition were more frequently linked to failure of amalgam restorations (26%) than composite restorations (5%), possibly due to the higher brittleness of amalgam under extreme occlusal forces [14].

The studies included in this review also highlighted variations in restorative techniques and operator experience, which could influence restoration longevity. Most studies utilized similar clinical protocols for both materials, including the use of local anesthesia, rubber dam isolation, and matrix placement techniques. However, differences in operator skill levels were observed, with some studies involving experienced clinicians and others including dental students. Studies with more experienced practitioners, such as those conducted by McCracken et al. (2013), demonstrated lower failure rates, underscoring the importance of technique sensitivity in composite resin restorations [12].

The methodological quality of the included studies varied, with most studies exhibiting moderate to high risk of bias. The primary source of bias was related to detection bias due to a lack of blinding in outcome assessments. Studies assessed using the NOS revealed that Opdam et al. (2010) had the highest methodological quality, while Al-Asmar et al. (2023) had a higher risk of bias due to moderate selection and exposure criteria scores [6,10,14]. The Cochrane ROB-2 tool identified selection bias as a minimal concern but highlighted moderate levels of performance and reporting bias across studies [7].

The findings of this systematic review reinforce the continued relevance of amalgam restorations in posterior permanent teeth, particularly in high-load areas where longevity is a primary concern. However, given the increasing aesthetic demands of patients and advancements in composite resin formulations, the use of composite restorations is expected to rise [23]. Strategies such as improved adhesive systems, bulk-fill composite techniques, and the incorporation of bioactive materials may enhance the longevity of composite restorations and close the durability gap with amalgam [24-26].

The present systematic review has certain limitations. Only studies published in the English language were included, which may have resulted in language bias and the exclusion of relevant data published in other languages. Additionally, while every effort was made to obtain complete datasets, a few studies had to be excluded due to missing or incomplete information, as attempts to contact corresponding authors were unsuccessful. Moreover, variability in study designs, follow-up durations, evaluation criteria, and outcome measures among the included studies may have introduced clinical and methodological heterogeneity, limiting the ability to make direct comparisons across studies. Lastly, although validated tools were used for risk of bias assessment, the inherent limitations of the primary studies, particularly observational ones, may influence the strength and generalizability of the conclusions drawn.

Further research is warranted to explore long-term performance outcomes of contemporary composite resins, particularly in large posterior restorations. Well-designed randomized controlled trials with extended follow-up periods, standardized failure criteria, and improved reporting of patient-related factors are needed to strengthen the evidence base. Additionally, future studies should consider the impact of emerging restorative techniques and materials, including nano-hybrid composites, resin-modified glass ionomer cements, and computer-aided design/computer-aided manufacturing (CAD/CAM)-based indirect restorations.

## Conclusions

Findings of the present systematic review highlight the superior longevity of amalgam restorations compared to composite resin restorations in posterior permanent teeth. The primary reasons for failure differ between the two materials, with secondary caries being more prevalent in composite restorations and fracture being the leading cause of amalgam restoration replacement. While patient preferences and aesthetic demands continue to drive the use of composite resins, clinicians must consider factors such as oral hygiene, occlusal forces, and restoration complexity when selecting the appropriate material. Ongoing advancements in composite resin technology and adhesive protocols may further enhance its longevity, providing a viable alternative to amalgam in the long term.

## Appendices

Appendix 1

Search Strategy for PubMed (via National Center for Biotechnology Information (NCBI) platform)

Date of last search: March 30, 2024 Database interface: NCBI PubMed (https://pubmed.ncbi.nlm.nih.gov/)

Search query:

("composite resins"[MeSH Terms] OR "composite resin"[All Fields] OR "resin composite"[All Fields] OR "direct composite"[All Fields]) AND ("dental restoration"[MeSH Terms] OR "restoration"[All Fields] OR "restorations"[All Fields]) AND ("repair"[MeSH Terms] OR "repair"[All Fields] OR "repaired"[All Fields]) AND ("replacement"[All Fields] OR "replaced"[All Fields] OR "replacement therapy"[MeSH Terms]) AND ("fracture"[All Fields] OR "fractured"[All Fields] OR "failure"[All Fields] OR "defective"[All Fields]) AND ("longevity"[All Fields] OR "survival"[All Fields] OR "durability"[All Fields] OR "success rate"[All Fields] OR "clinical outcome"[All Fields]) AND ("permanent teeth"[MeSH Terms] OR "permanent tooth"[All Fields] OR "adult dentition"[All Fields])

Filters applied:

Language: English

Species: Humans

Study types: Clinical trials, Observational studies, Cohort studies, Randomized controlled trials
